# Industrial Efficiency and Eyesight

**Published:** 1916-12-23

**Authors:** 


### The Hospital

The Workers' Newspaper of Administrative Medicine and Institutional Life, Administration, National Insurance and Health.

No. 1594, Vol. LXI. SATURDAY, DECEMBER 23, 1916. PRICE ONE PENNY

### INDUSTRIAL EFFICIENCY AND EYESIGHT.

The relations inevitably existing between health and industrial efficiency are vivid enough to the individual household when disease or disability strikes the breadwinner. Such a position, too, makes an appeal to a more or less limited circle of outsiders, but these, and the rest, so far as they learn the facts, regard the occasion mainly as one of misfortune for the worker and those dependent on his earnings. To think of this disaster as an example of many and to contemplate the effect of the sum of these upon the efficiency and position of the nation was formerly an exercise reserved to statisticians and professors of political economy. But nowadays the nation as a whole comes under the review of most of us, and we begin to realise that whatever acts prejudicially on one class may well put the interests of all in peril. Our neighbour is not only our neighbour. He is part and parcel of the machinery by which national safety is secured, and his collapse may help to bring to a stop the working of an organisation behind which we all hope for safe shelter. Health and efficiency have thus become conspicuous as a national asset of the first order. The display of this truth has been specially prominent in the case of the Army, and has led to a correspondingly wide recognition of the work and responsibility of the medical officers, upon whom the fighting ranks depend for fit and active soldiers. The casualty lists demand attention, of course, but even more important in the national interest is the operation of measures by which the armies are protected from disease and are maintained in full physical vigour and fitness. Now it is being gradually recognised that the same truths apply to the health of the industrial workers, whose place it is to furnish the deadly weapons and munitions without which victory is impossible. Here also prevention takes the first rank, and, in so far as this fails, it is not the mere suffering of individuals which is at stake, but also the national interest. The statement, when once made, is admitted as a commonplace, but the acuteness of the present hour has proclaimed it in large letters, so that even he who runs may read. The health of the working community is seen as a large element in the combination necessary to the success of the nation's cause.

The propositions here stated afford an explanation of the existence of the official Committee charged to consider the health of men and women engaged in the manufacture of munitions. Under the chairmanship of Sir George Newman, the Committee has already issued a number of valuable reports, and to some of these attention has been duly directed in these columns. The latest of the series is one dealing with the eyesight of the workers. Like previous reports, it includes a carefully gathered collection of facts and recommendations based on these facts. It thus affords a further example of the linking of a body of expert knowledge to the purposes of the war, and incidentally it may be expected to increase the appreciation of the closeness of the relation between individual health and national efficiency. The lesson is read to us in the terrible notes of war, but one may hope that its application to the victories of peace will also be kept in mind.

As might well be expected, the increase in the number of workers and their want of familiarity with the tasks committed to them have led to a considerable increase in the number of injuries of the eye dealt with in the general and special hospitals in various parts of the country. The same remark applies to conditions which result from eye-strain, for in some parts of the work fine manipulation and delicate measurements are essential, and these mean efforts towards keen vision. It is the case, fortunately, that most of the injuries inflicted, as by minute flying particles, are slight, but when the aggregate number is contemplated, the total loss of time which results is substantial, and this in its turn means diminution of output, with its necessary prejudice to the national effort. Still more important is it to read that for want of prompt and efficient treatment many of these slight and superficial injuries become septic, with obviously great risk to the individual and with a further lessening of the number of active workers. Such results, bad as they are in themselves, assume an aggravating character when one learns that in a very large measure these accidents are preventable. There is not only loss to the nation, but also loss which might be avoided. The advice given by the Committee does not contain anything which is new to ophthalmic surgeons who practise in industrial communities, but the special circumstances in which it is given may perhaps secure greater attention to it. Lathe-turning plays a large part in munition-making, as it does in more peaceful occupations, and from it arise many of the cases of small foreign bodies embedded in the eyeball. For various reasons the workers dislike the continued use of protective goggles, which is the most radical measure of prevention, and the Committee has considered these objections with a view to overcome them. There are certain classes of work in which such guards should always be worn, but it may be doubted whether anything short of compulsion will secure their adoption. Again, workers must be taught that no injury to the eye, however slight it may appear, can be safely treated except under medical direction, and that some of the amateur methods which are practised are full of peril, inasmuch as they involve the risk of sepsis, with the danger of loss of vision or even of the eyeball. Another measure of protection is that all those engaged in anything like fine work should before entering on their duties be thoroughly examined by an ophthalmic surgeon, and that those who suffer from errors of refraction should be compelled to wear appropriate glasses. These counsels, we repeat, are not new; but they are timely. It is for those who carry responsibility to see that the Committee's recommendations are applied in practice, and in doing so they will be helping to strengthen a weak place in the scheme of national defence.

